# NMDA GluN2C/2D receptors contribute to synaptic regulation and plasticity in the anterior cingulate cortex of adult mice

**DOI:** 10.1186/s13041-021-00744-3

**Published:** 2021-03-25

**Authors:** Qi-Yu Chen, Xu-Hui Li, Jing-Shan Lu, Yinglu Liu, Jung-Hyun Alex Lee, Yu-Xin Chen, Wantong Shi, Kexin Fan, Min Zhuo

**Affiliations:** 1 Center for Neuron and Disease, Frontier Institutes of Science and Technology, Xi’an Jiaotong University, Xi’an, China; 2 International Institute for Brain Research, Qingdao International Academician Park, Qingdao, China; 3 Department of Physiology, University of Toronto, 1 King’s College Circle, Toronto, ON Canada

**Keywords:** NMDAR, GluN2C/2D, ACC, LTP, LTD

## Abstract

**Introduction:**

N-Methyl-D-aspartate receptors (NMDARs) play a critical role in different forms of plasticity in the central nervous system. NMDARs are always assembled in tetrameric form, in which two GluN1 subunits and two GluN2 and/or GluN3 subunits combine together. Previous studies focused mainly on the hippocampus. The anterior cingulate cortex (ACC) is a key cortical region for sensory and emotional functions. NMDAR GluN2A and GluN2B subunits have been previously investigated, however much less is known about the GluN2C/2D subunits.

**Results:**

In the present study, we found that the GluN2C/2D subunits are expressed in the pyramidal cells of ACC of adult mice. Application of a selective antagonist of GluN2C/2D, (2R*,3S*)-1-(9-bromophenanthrene-3-carbonyl) piperazine-2,3-dicarboxylic acid (UBP145), significantly reduced NMDAR-mediated currents, while synaptically evoked EPSCs were not affected. UBP145 affected neither the postsynaptic long-term potentiation (post-LTP) nor the presynaptic LTP (pre-LTP). Furthermore, the long-term depression (LTD) was also not affected by UBP145. Finally, both UBP145 decreased the frequency of the miniature EPSCs (mEPSCs) while the amplitude remained intact, suggesting that the GluN2C/2D may be involved in presynaptic regulation of spontaneous glutamate release.

**Conclusions:**

Our results provide direct evidence that the GluN2C/2D contributes to evoked NMDAR mediated currents and mEPSCs in the ACC, which may have significant physiological implications.

## Introduction

As a key glutamate-gated ion channel in the central nervous system, N-Methyl-d-aspartate receptors (NMDARs) attract a lot of attention because of their known roles in synaptic plasticity [1, 2]. Subunits of NMDARs are classified in three main families which are identified according to the homogeneity: GluN1, GluN2A/2B/2C/2D and GluN3A/3B. Structural studies have already reported that NMDARs are assembled in tetrameric form, in which two GluN1 subunits and two GluN2 and/or GluN3 subunits combine together as di-heteromeric or tri-heteromeric receptors. The subunit composition varies during neurodevelopment, and determines channel properties of NMDARs [3]. Among all the subunits, NMDARs with GluN2C/2D subunits display lower sensitivity to Mg^2+^ ions and slower deactivation kinetics compared with GluN2A/2B containing NMDARs. GluN2C/2D-contaning NMDARs could allow selective modification of circuit function in regions expressing GluN2C/2D subunits[4–7]. The expression of GluN2C/2D has been reported in adult cerebral cortices [8–13]. GluN2D subunits were reported to play roles in synaptic transmission of hippocampal neurons[14, 15]. The activation of NMDARs by glutamate spillover was prevented by the GluN2D-selective antagonists.

In the anterior cingulate cortex (ACC), a critical cortical region involved in chronic pain and emotions, the roles of NMDAR subunits in synaptic plasticity have been investigated [2, 16, 17]. Application of the NMDAR antagonist AP-5 blocked both the NMDAR- mediated EPSCs and the induction of the postsynaptic form of long-term potentiation (post-LTP). Both application of the GluN2A antagonist PEAQX and of GluN2B antagonist ifenprodil or Ro 25-6981 blocked the NMDAR- mediated EPSCs, which indicates NMDAR containing GluN2A or GluN2B subunits contribute to most of the NMDAR currents [18]. The application of antagonist also reduced, or completely abolished (co-application of both inhibitors) the post-LTP. GluN2A and GluN2B were also proved to be required for long-tern depression (LTD) in the ACC [19]. Aside from the focus on the GluN2A/2B, genes encoding GluN2C and GluN2D have also been detected by in-situ hybridization in the ACC of mice (http://mouse.brain-map.org/experiment/show/75888748, http://mouse.brain-map.org/experiment/show/75551479). GluN2C and GluN2D were detected in the ACC of humans by western blot [20], but less studies have been carried out on the function of GluN2C/2D containing NMDARs in the ACC of mice.

Therefore, in this study, we first detected the expression of GluN2C/2D in the ACC of adult mice by Western blot. Then we performed whole-cell patch-clamp recordings from the brain slices of the ACC in adult mice. By using a selective antagonist of GluN2C/2D, (2R*,3S*)-1-(9-bromophenanthrene-3-carbonyl) piperazine-2,3-dicarboxylic acid (UBP145), we found that the GluN2C/2D modulates synaptic transmission in a presynaptic pattern, and they partially composed the NMDAR-mediated EPSCs in pyramidal neurons of the ACC. Our results indicate that GluN2C/2D may also be involved in the functions of NMDARs in the ACC.

## Material and methods

### Animals

Adult male C57BL/6 mice (7–9 weeks old) were used. All animals were housed under a 12 h light/dark cycle with food and water provided ad libitum. Experiments were conducted under the protocol approved by the Ethics Committee of Xi’an Jiaotong University and the University of Toronto.

### Drugs

The chemical and drugs used in this study were as follows: AP-5, CNQX and Ro 25-6981 purchased from HelloBio (Princeton, NJ, USA), PPDA, UBP145 and PEAQX were purchased from Tocris Cookson (Bristol, UK), PTX was bought from Sigma-Aldrich (St Louis, MO, USA). Drugs were prepared as stock solutions for frozen aliquots at -20℃. All drugs were diluted from the stock solution to the final desired concentration in the ACSF before being applied to the perfusion solution.

### Brain slices preparation

Coronal brain slices (300 μm) of ACC were prepared using standard methods. Briefly, mice were deeply anesthetized with 5% isoflurane and sacrificed by decapitation. The whole brain was removed quickly from the skull and submerged in the oxygenated (95% O_2_ and 5% CO_2_) ice-cold artificial cerebrospinal fluid (ACSF) containing (in mM) 124 NaCl, 2.5 KCl, 2 MgSO_4_, 1 NaH_2_PO_4_, 2 CaCl_2_, 25 NaHCO_3_, and 10 D-glucose. The whole brain tissue was cooled for short time before the tissue containing the target region was isolated and glued onto the vibratome (VT1200S Vibratome, Leica, Germany). Slices were incubated in a submerged recovery chamber at room temperature for one hour. The ACSF was continuously balanced with a mixture of 95% O_2_ and 5% CO_2_.

### Western blot

Brain regions were homogenized in 10 mM Tris (pH 7.4), 2 mM EDTA, and 1% SDS, 1× protease inhibitor cocktail. Lysates were centrifuged at 14,000 rpm, 4 °C for 20 min, and the supernatants were used for protein analysis and Western blotting. Equal amounts (40–50 μg) of protein from tissue lysates were separated by SDS-PAGE and transferred to polyvinylidene difluoride membrane followed by blocking with 5% skim milk for 1 h at room temperature. Blots were probed with anti-GluN2A (1:1000; Millipore, US), anti-GluN2B (1:500; Millipore, US), anti-GluN2C (1:500; Millipore, US) or anti-GluN2D (1:500; Millipore, US) polyclonal antibodies overnight at 4 °C. For tubulin blotting as a control, a monoclonal antibody (1:4000; Sigma, US) was used. The membranes were incubated with a horseradish peroxidase-conjugated goat anti-rabbit and anti-mouse IgG (1:5000; Millipore, US) for 1 h at room temperature, and the bands were visualized by an ECL system (GE Healthcare, US). Exposure time varied for each primary antibody to ensure that the signals were in the linear range. Signals were quantified using ImageJ software.

### Whole-cell patch-clamp recording

Whole cell recordings were performed in a recording chamber on the stage of an Olympus BX51 microscope with infrared differential interference contrast (DIC) optics for visualization. EPSCs were recorded from layer II/III neurons with an Axon 200B amplifier (Molecular Devices), and the stimulations were evoked in layer V of the ACC by a bipolar tungsten stimulating electrode. The recording pipettes (3–5 MΩ) were filled with the solution containing (in mM) 145 K-gluconate, 5 NaCl, 1 MgCl_2_, 0.2 EGTA, 10 HEPES, 2 Mg-ATP, and 0.1 Na_3_-GTP, which adjusted to pH 7.3 with KOH and had osmolality of 300 mOsmol. The amplitudes of evoked EPSCs were adjusted to between 100 and 150 pA to obtain a baseline. When recording NMDAR-mediated EPSCs, a holding potential of + 30 mV was used as indicated. CNQX (20 µM) was added into the perfusion solution. Cs-gluconate was used to replace the K-gluconate. For miniature EPSCs (mEPSCs) recordings, tetrodotoxin (TTX, 1 µM) was added into the perfusion solution. Picrotoxin (PTX, 100 μm) was always present to block the GABA_A_ receptor-mediated inhibitory synaptic currents in all experiments. Access resistance was 15–30 MΩ and monitored throughout the experiment. Data was collected only when access resistance changed < 15% during all experiments. Data was filtered at 1 kHz, and digitized at 10 kHz. The synaptic potentiation was observed according to previous reports[18, 19, 21]. After obtaining stable EPSCs for 5 to 10 min as baseline, postsynaptic LTP was induced by paired presynaptic 80 pulses at 2 Hz with postsynaptic depolarization at + 30 mV. LTD was induced by 300 pulses at 1 Hz paired with postsynaptic depolarization at − 45 mV. Presynaptic LTP was induced by low-frequency stimulation (2 Hz, 2 min). After stimulation, the test stimulus was repeatedly delivered once every 30 s to monitor the time course of LTP or LTD.

### Statistical analysis

OriginPro 8.0 (Originlab Corporation, Northampton, MA) was used for plotting figures and SPSS version 22.0 (SAS Institute Inc, Cary, NC) software was used to analyze the results. The paired t-tests or one-way ANOVA was conducted as appropriate. All data were presented as the mean ± standard error of the mean (SEM). In all cases, p < 0.05 was considered statistically significant.

## Results

### NMDAR GluN2A-D subunits expressed in both the ACC and the hippocampus

**Fig. 1:**
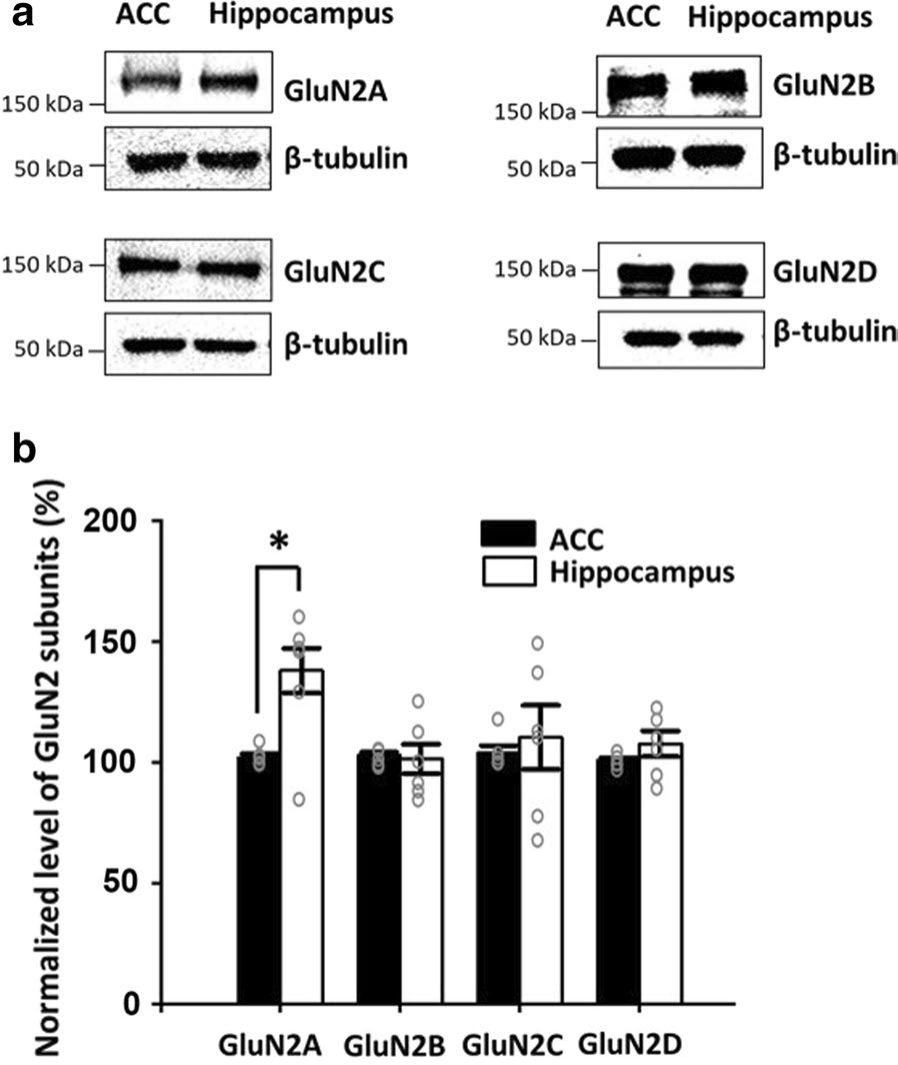
NMDAR GluN2A-D subunits expressed in both the ACC and the hippocampus. **a** Representative Western blots of NMDAR subunit GluN2A-2D expression in total homogenates of the ACC and hippocampus. **b** Percentage of GluN2A-GluN2D in the ACC (black) and hippocampus(white) (n = 6 in each group). Open circles represent the individual data points. *p < 0.05, versus the ACC. Error bars represent SEM

As an essential part of NMDAR subunit components, GluN2A-D subunits have received great attention recently. Previous studies report that GluN2A-B contributes to NMDAR mediated responses in the ACC [18, 22], less is known about GluN2C and GluN2D subunits in the ACC of mice. By using Western blot, we wanted to determine if GluN2C/2D can be detected in the ACC of adult mice. We found that both GluN2C and GluN2D subunits were expressed in the ACC of adult mice (Fig. 1a). For comparison, we also measured the expression of the same subunits in the hippocampus of the same animals. Similar to previous reports [18], we confirmed that the basal expression level of GluN2A subunits was consistently higher in the hippocampus compared to the ACC. In contrast, GluN2B subunit expression was similar in both the ACC and the hippocampus (Fig. 1b).

Furthermore, we compared the expression level of GluN2C and GluN2D subunits between the ACC and hippocampus. We found that the expression level of GluN2C and GluN2D subunits had no significant difference between the ACC and hippocampus (GluN2C: ACC 103.8 ± 3.1% vs. hippocampus 110.3 ± 13.3%, paired *t*-test, *p* = 0.62; GluN2D: ACC 100.7 ± 1.0% vs. hippocampus 107.7 ± 5.4%, paired *t*-test, *p* = 0.88, n = 6 mice in each group, Fig. 1b). These results indicate that GluN2C and GluN2D subunits are present in the ACC of adult mice, which may play a role in synaptic transmission or synaptic plasticity.

## The GluN2C/2D components of the NMDAR-mediated EPSCs in the ACC

**Fig. 2:**
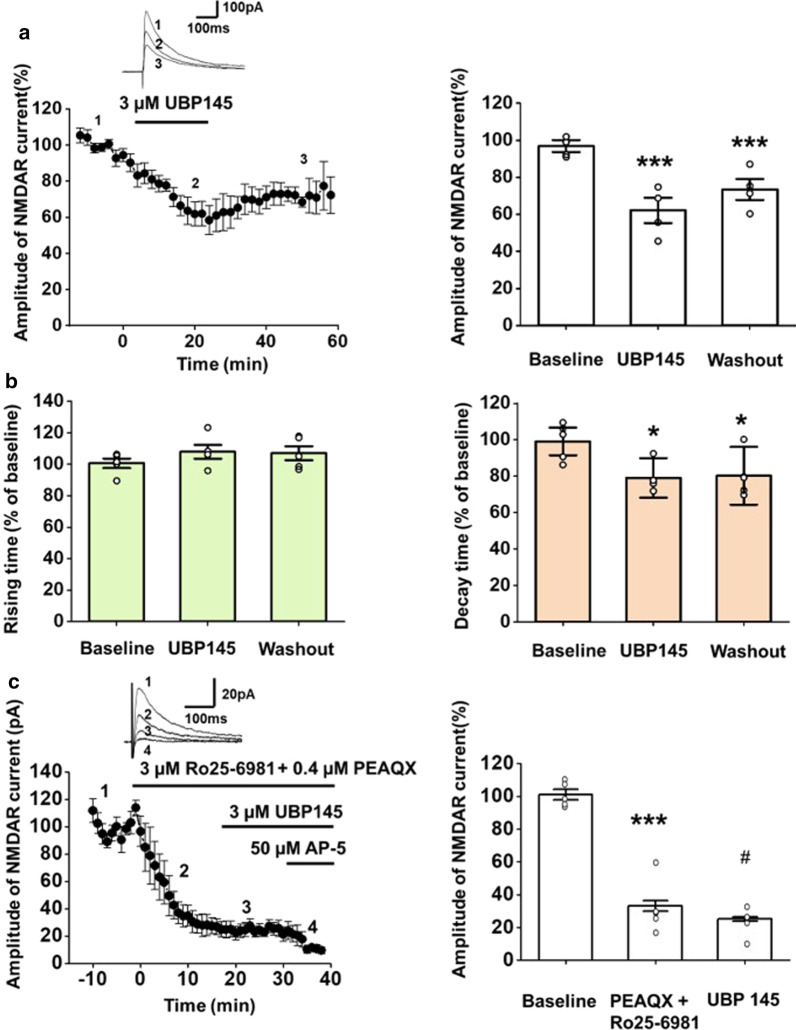
The GluN2C/2D components of the NMDAR-mediated EPSCs are significant in neurons of the ACC by using UBP145 as the antagonist of GluN2C/2D. **a** A selective GluN2C/2D antagonist, UBP145, partially inhibited NMDAR-mediated EPSCs. Left: the time course of changes in EPSC amplitude before, during and after the application of UBP145 (3 μM) in ACC neurons from adult male mice is shown. Traces show the currents at different time points during application of drugs. UBP145 produced its maximal effect at 20 min after bath application and was partially washed out (n = 5 neurons/3 mice). Right: Summary of the partial inhibition of the UBP145 on the NMDAR-mediated current. Data within the last five minutes of the baseline, UBP145 application and washout phase is averaged. Open circles represent the individual data points. **b** Summarized data of the effect of UBP145 to the rising time (left) and decay time (right) of the NMDAR-mediated EPSCs in the ACC neurons. The application of UBP145 produced a significant inhibitory effect on the decay time of EPSCs. **c** Left: application of the antagonist of the GluN2A (PEAQX) and GluN2B (Ro 25-6981) reduced over 60% of the NMDAR current. UBP145 further decreased the amplitude of EPSC. The remaining current were gradually decreased by the application of AP-5. Right: Summary of the gradual inhibition of the GluN2A/2B and GluN2C/2D on the NMDAR-mediated current. * *p* < 0.05, *** *p* < 0.001, compared with baseline; # *p* < 0.05, compared with PEAQX + Ro25-6981. Error bars represent SEM

The GluN2A/2B components of the NMDAR-mediated EPSCs in pyramidal neurons of the ACC have been dissected by using selective antagonists of the NMDAR subunits [22]. NMDAR-mediated EPSCs were isolated by applying AMPA/KA receptor antagonist CNQX and GABA_A_ receptor antagonist picrotoxin. Here, we used a selective antagonist of GluN2C/2D, UBP145 to investigate the GluN2C/2D components of the NMDAR-mediated EPSCs in the ACC. The amplitude of NMDAR- mediated EPSCs reduced to 62.1 ± 6.8% of baseline after the administration of UBP145 for 20 min (one-way ANOVA, *F*_(2,12)_ = 206.4, *p* < 0.001, Fig. 2a, b). The effect of UBP145 was slightly recovered to 73.4 ± 5.7% of baseline after being washed out for 40 min (Fig. 2a). Since one of the functional features of the GluN2C/2D is slowly deactivated kinetics, we observed that the decay time of the NMDAR-mediated EPSCs significantly decreased after the administration of UBP145 (79.1 ± 10.8% of baseline, *F*_(2,12)_ = 5.6, *p* = 0.019, n = 5 neurons/3 mice, Fig. 2b) while the rising time remained at 107.9 ± 4.4% of baseline. To test if UBP145 may affect non GluN2C/2D currents, we applied the combination of GluN2A (PEAQX) and GluN2B (Ro25-6981) antagonists to block the components of GluN2A/2B first, and then tested the effect of UBP145. We found that the amplitude of NMDAR-mediated EPSC was decreased (33.2 ± 7.6% of baseline, LSD *t*-test, *p* < 0.001, n = 5 neurons/3 mice, Fig. 2c) by the inhibition of GluN2A and 2B. Subsequent application of UBP145 caused further reduction of the amplitude (mean 25.2 ± 4.6% of baseline, LSD *t*-test, *p* = 0.014, n = 5 neurons/3 mice, Fig. 2c). The rest of the NMDAR- mediated current was reduced by the final application of the NMDAR antagonist AP-5.

**Fig. 3:**
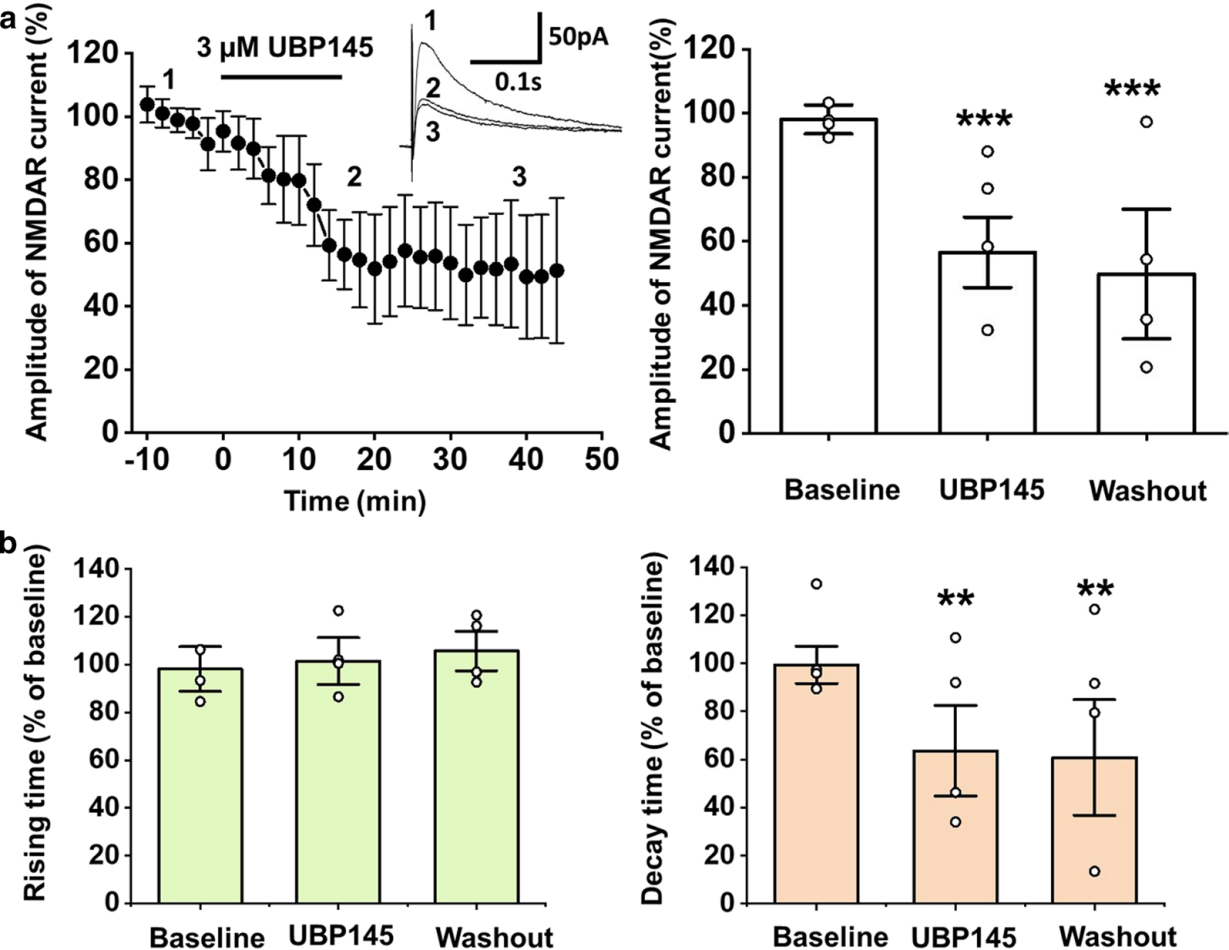
The GluN2C/2D components of the NMDAR-mediated EPSCs are significant in neurons of the CA1 by using UBP145 as antagonist of GluN2C/2D. **a** UBP145 partially inhibited NMDAR-mediated EPSCs in the CA1. Left: traces show the currents at different time points during application of drugs. UBP145 produced its maximal effect at 20 min after bath application (n = 4 neurons/3 mice). Right: Summary of the partial inhibition of the UBP145 on the NMDAR-mediated current. Data within the last five minutes of the baseline, UBP145 application and washout phase is averaged. Open circles represent the individual data points. **b** Summarized data of the effect of UBP145 to the rising time (left) and decay time (right) of the NMDAR-mediated EPSCs in the CA1 neurons. The application of UBP145 produced a significant inhibitory effect on the decay time of EPSCs. **p < 0.01. ***p < 0.001. Error bars represent SEM

Since the presence of GluN2C/2D has also been reported in hippocampus, we also measured the GluN2C/2D components of the NMDAR-mediated EPSCs in the CA1 of adult mice for comparison. The amplitude of NMDAR- mediated EPSCs reduced to 56.5 ± 11.0% of baseline 20 min after the administration of UBP145 (one-way ANOVA, *F*_(2,12)_ = 250.6, *p* < 0.001, n = 4 neurons/3 mice, Fig. 3). The decay time of the NMDAR-mediated EPSCs decreased after UBP145 was applied (*F*_(2,12)_ = 13.3, *p* = 0.001, n = 4 neurons/3 mice). These results further confirm that the GluN2C/2D contributes to the synaptic modulation in both the ACC, and the hippocampus.

### Inhibiting GluN2C/2D neither altered the electrically-evoked responses nor the paired-pulse ratio

**Fig. 4:**
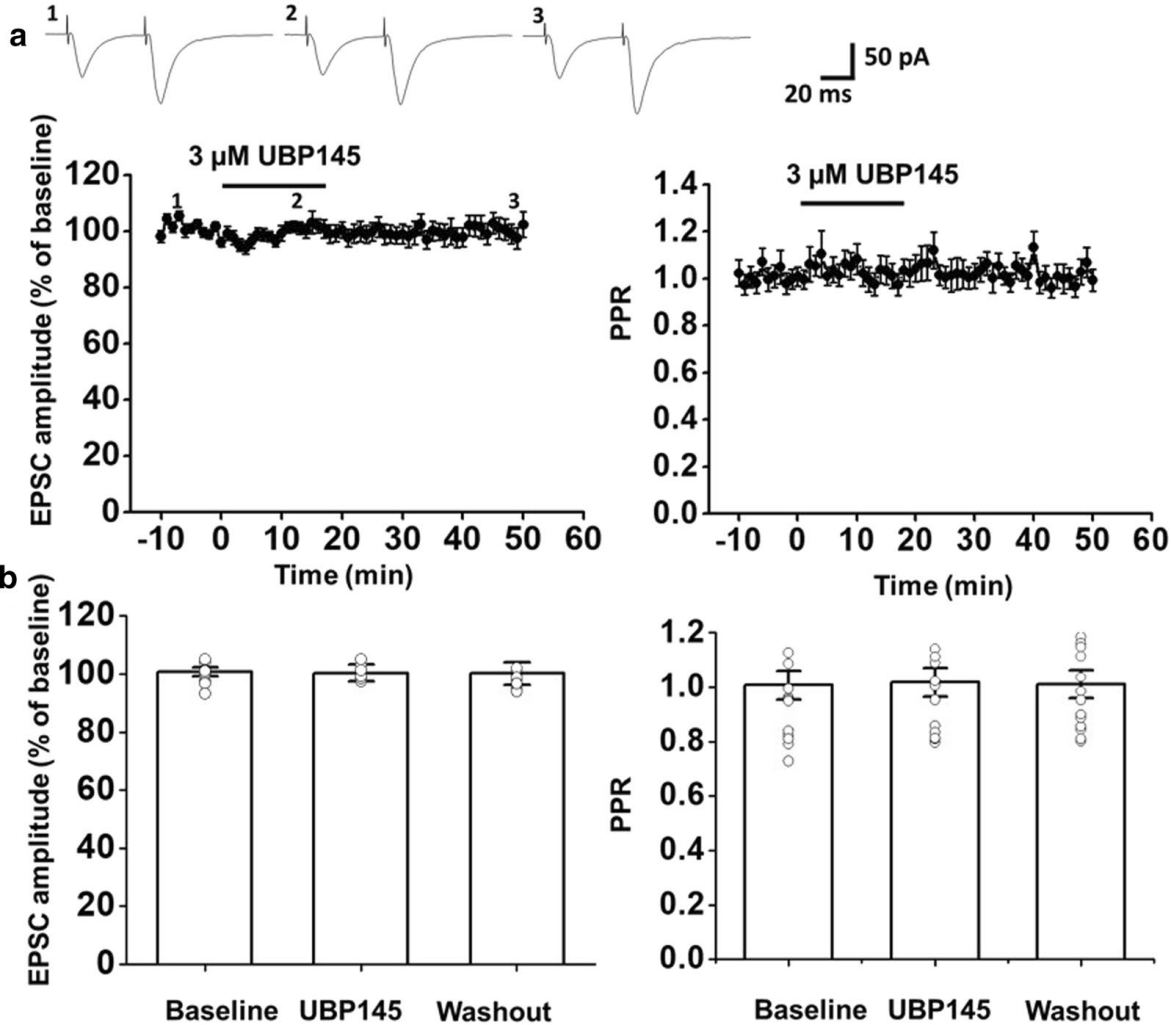
UBP145 was not able to change the evoked responses. **a** Pooled data illustrated the UBP145 neither has significant effect on the evoked response (left, filled circles) nor the paired-pulse ratio (PPR, right, filled triangles) when the membrane potential was patched at - 70 mV (n = 6 neurons/5 mice). Sample traces showed the pair-pulse responses at different time course before, during and after the application of UBP145. **b** Summary of the effect of the UBP145 on the NMDAR-mediated current. Data within the last five minutes of the baseline, drug application and washout phase are averaged. Open circles represent the individual data points. Error bars represent SEM

To investigate the role of GluN2C/2D in evoked responses, we kept the holding potential at -70 mV and recorded the responses for paired-pulse stimulation. We found that the amplitude did not show significant change (100.4 ± 2.9% of baseline, one-way ANOVA, *F*_(2,12)_ = 0.8, *p* = 0.48, n = 6 neurons/5 mice, Fig. 4a) when the UBP145 was applied, as well as the paired-pulse ratio (1.6 ± 0.3 compared with baseline 1.01 ± 0.05, *F*_(2,12)_ = 0.65, *p* = 0.54, Fig. 4). These results indicated that UBP145 was not able to change the basic evoked transmission. Meanwhile, the evoked presynaptic release was not altered by inhibiting GluN2C/2D.

### GluN2C/2D did not participate in the synaptic plasticity in the ACC

**Fig. 5:**
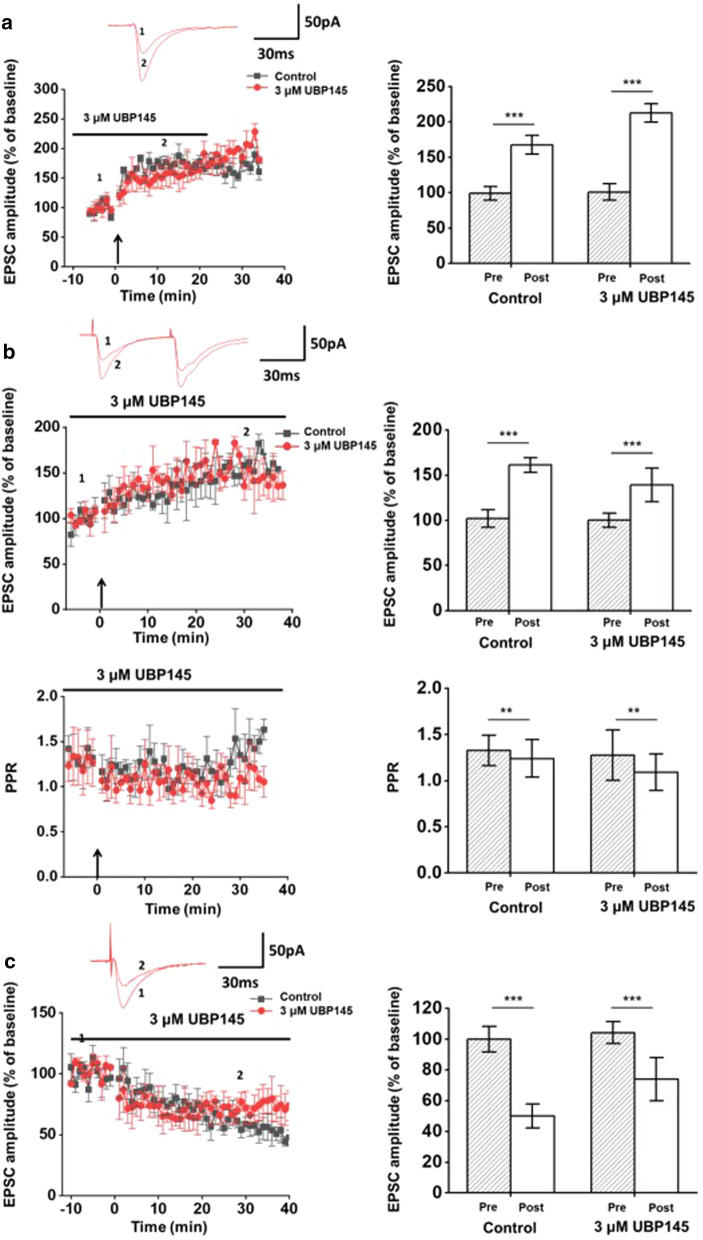
Effects of GluN2C/2D antagonists to the synaptic plasticity in the ACC. **a **Left: LTP was induced in pyramidal neurons in adult ACC (black squares, n = 5 neurons/ 3 mice) by the pairing training protocol (indicated by an arrow). Pretreated slices with 3 μM UBP145 didn’t affect the induction of LTP (red circles, n = 4 neurons/ 3 mice). Sample traces of evoked EPSCs during baseline (1) and 30 min after the induction stimulus (2) are showed on the top; Right: Summary of the effect of the UBP145 on the postsynaptic LTP. Data within the last five minutes of the baseline and 30 min after the induction are averaged. **b **Top: pre-LTP was induced in pyramidal neurons in the ACC of adult mice (black squares, n = 4 neurons/ 3 mice) by giving 240 pulses at 2 Hz (indicated by an arrow). Pretreated slices with 3 μM UBP145 did not affect the induction of pre-LTP (red circles, n = 4 neurons/ 3 mice). Sample traces of evoked EPSCs with paired-pulse stimulation at 50 ms at a holding membrane potential of -70 mV during baseline (1) and 30 min after the induction stimulus (2) are showed on the top; Bottom: PPR values for the control (black squares) and UBP145 group (red circles). **c** LTD was induced in pyramidal neurons in adult ACC (black squares, n = 5 neurons/ 3 mice) by the pairing training protocol (300 pulses at 1 Hz while holding at -45 mV indicated by an arrow). Pretreated slices with 3 μM UBP145 did not affect the induction of LTD (red circles, n = 4 neurons/ 3 mice). Sample traces of evoked EPSCs during baseline (1) and 30 min after the induction stimulus (2) are showed on the top. **p < 0.01, ***p < 0.001. Error bars represent SEM

To investigate roles of GluN2C/2D in synaptic plasticity in the ACC, we added UBP145 into the ACSF and incubated the slices in the drugs for at least 15 min before the recording. After maintaining a stable baseline for 5 min, we induced post-LTP in the ACC according to protocols that are reported in our pervious papers [18]. We found that UBP145 did not block post-LTP (Control: 167.6 ± 3.3% of baseline, n = 5 neurons/3 mice; UBP145: 216.7 ± 16.4% of baseline, n = 4 neurons/3 mice, *p* < 0.001, Fig. 5a). Results of UBP145 demonstrate that GluN2C/2D may not be required for the induction of post-LTP in the ACC.

In the ACC, pre-LTP has also been discovered and is considered to be related to anxiety [21]. Here we tried to study whether the GluN2C/2D is involved in the pre-LTP. We found that application of UBP145 did not block the induction of pre-LTP in the ACC as well as the decrease of the paired-pulse ratio. (Control: 161.4 ± 8.2% of baseline, n = 4 neurons/3 mice; UBP145: 139.5 ± 18.5% of baseline, n = 4 neurons/3 mice, *p* < 0.001, Fig. 5b). These results demonstrate that GluN2C/2D may not be involved in the induction of presynaptic LTP in the ACC.

In the ACC, NMDARs, especially GluN2A and GluN2B are also found to be important for LTD [2, 19]. Therefore, we investigated whether GluN2C/2D modulate LTD in the ACC. 10 min after the UBP145 was applied, LTD was induced by 300 pulses at 1 Hz while holding at -45 mV. We found that inhibiting GluN2C/2D didn’t block the LTD in the ACC (Fig. 5c). These results demonstrate that GluN2C/2D may not be involved in the induction of LTD in the ACC.

### GluN2C/2D modulate presynaptic release in the ACC

**Fig. 6:**
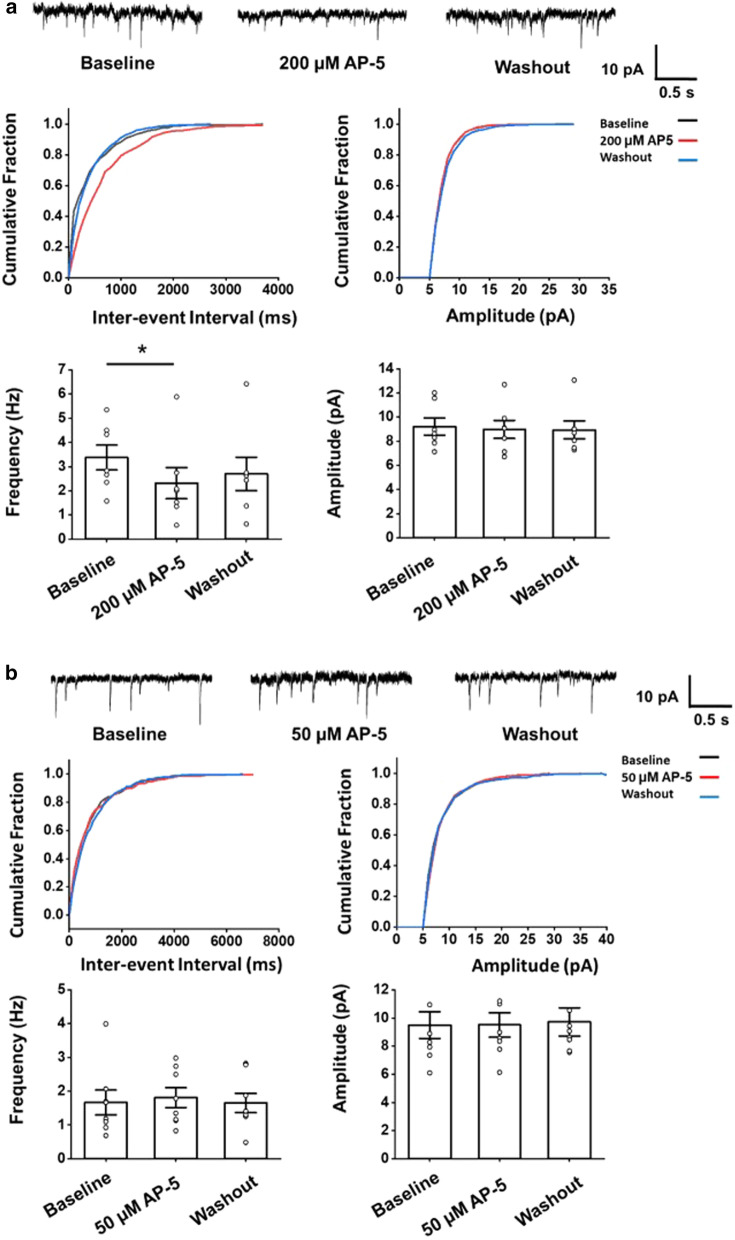
NMDAR modulates spontaneous presynaptic release in the ACC. **a** Top: Representative traces of the mEPSCs recorded in the ACC neurons before and after applying a high dose of AP-5 (200 μM). Middle: Cumulative fraction of inter-event interval (left) and amplitude (right) of the mEPSCs in the phase of baseline (black line), AP-5 application (red line) and wash out (blue line). Bottom: Statistic results of the frequency (left) and amplitude (right) of mEPSCs (n = 9 neurons/6 mice). **b** Top: Representative traces of the mEPSCs recorded in the ACC neurons before and after applying a lower dose of AP-5 (50 μM). Middle: Cumulative fraction of inter-event interval (left) and amplitude (right) of the mEPSCs in the phase of baseline (black line), AP-5 application (red line) and wash out (blue line). Bottom: Statistic results of the frequency (left) and amplitude (right) of mEPSCs (n = 9 neurons/6 mice). Open circles represent the individual data points. *p < 0.05, error bars indicated SEM

Our previous studies showed that GluN2A and GluN2B are required for the excitatory postsynaptic responses in the ACC. Here, we investigate how GluN2C/2D affects synaptic transmission. We recorded mEPSCs of the pyramidal neuron in the ACC of adult male C57 mice. After applying the antagonist of NMDAR, AP-5, at a high dose (200 μM), the frequency of mEPSCs significantly decreased compared with the baseline period (3.4. ± 0.5 Hz vs. 2.3 ± 0.6 Hz; paired *t*-test, *p* = 0.023; n = 7 neurons/4 mice), while the amplitude did not show a significant change (9.2 ± 0.7 pA vs. 9.0 ± 0.7 pA; *p* = 0.60) (Fig. 6a). A lower dose (50 μM) AP-5 had no significant effect on the mEPSCs (Frequency: 1.6 ± 0.4 Hz vs. 1.8 ± 0.3 Hz; *p* = 0.56; Amplitude: 9.5 ± 1.0 pA vs. 9.5 ± 0.9 pA; *p* = 0.98; n = 8 neurons/4 mice, Fig. 6b). These results indicate that NMDAR modulates the presynaptic glutamate release in the ACC.

**Fig. 7:**
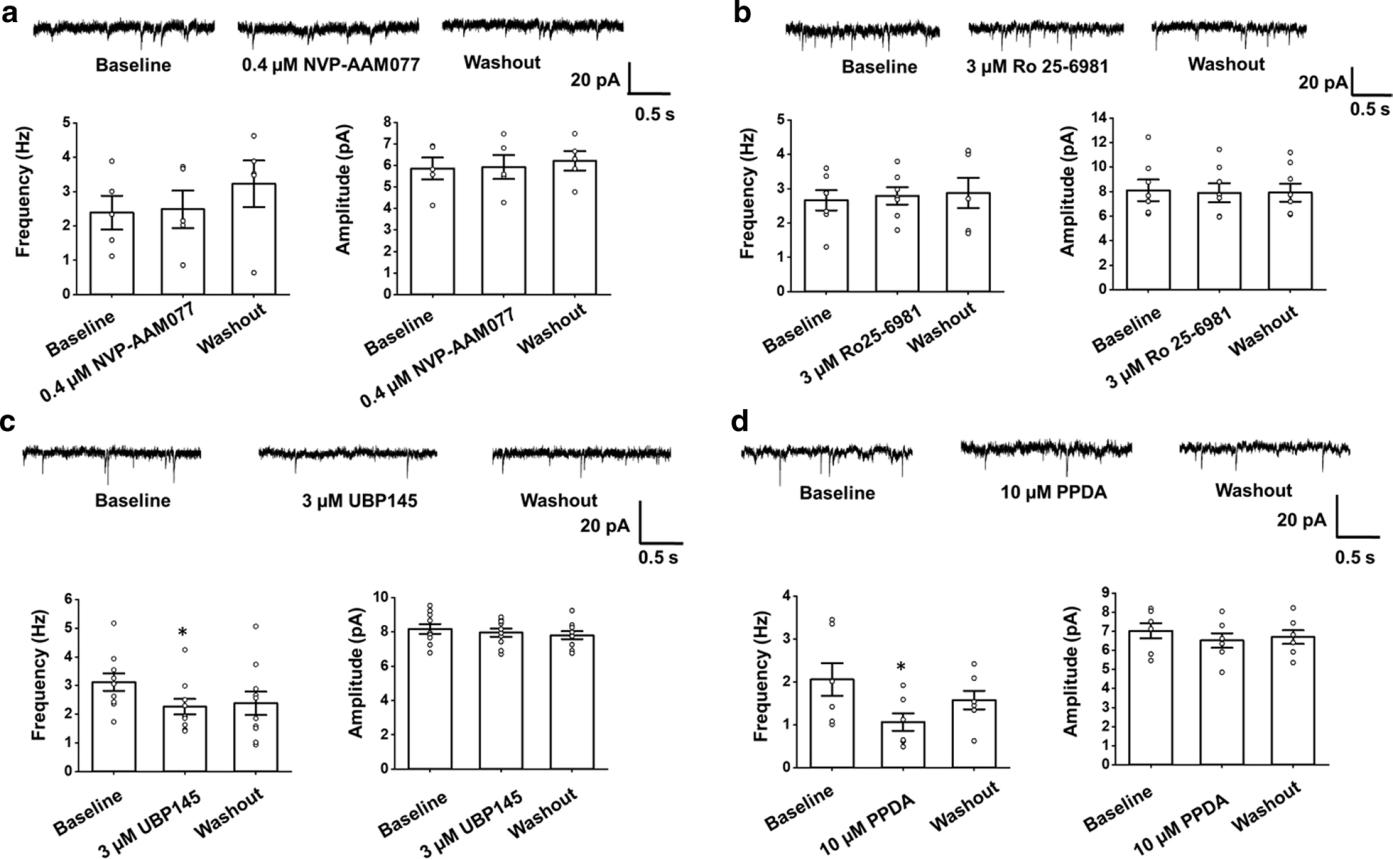
GluN2C/2D modulate spontaneous presynaptic release in the ACC. **a** Top: Representative traces of the mEPSCs recorded in the ACC neurons before and after applying PEAQX (0.4 μM), a selective antagonist of GluN2A. Bottom: Statistic results of the frequency (left) and amplitude (right) of mEPSCs (n = 5 neurons/3 mice). **b** Top: Representative traces of the mEPSCs recorded in the ACC neurons before and after applied Ro 25-6981 (3 μM), a selective antagonist of GluN2B. Bottom: Statistic results of the frequency (left) and amplitude (right) of mEPSCs (n = 7 neurons/6 mice). **c** Top: Representative traces of the mEPSCs recorded in the ACC neurons before and after applying UBP145 (3 μM). Bottom: Statistic results of the frequency (left) and amplitude (right) of mEPSCs (n = 10 neurons/4 mice). **d** Top: Representative traces of the mEPSCs recorded in the ACC neurons before and after applied PPDA (10 μM). Bottom: Statistic results of the frequency (left) and amplitude (right) of mEPSCs (n = 5 neurons/3 mice). Open circles represent the individual data points. *p < 0.05, error bars indicated SEM

To further investigate which subunit is involved in this process, we used UBP145 and another antagonist of GluN2C/2D, PPDA, to take the place of AP-5. The frequency of mEPSCs significantly decreased after applying UBP145 or PPDA (UBP145: 3.1 ± 0.3 Hz vs. 2.3 ± 0.3 Hz; paired *t*-test, *p* = 0.038, n = 10 neurons/4 mice; PPDA: 2.2 ± 0.4 Hz vs. 1.0 ± 0.2 Hz; *p* = 0.012; n = 5 neurons/3 mice), while the amplitude did not change significantly (UBP145: 8.2 ± 0.3 pA vs. 8.0 ± 0.2 pA; *p* = 0.57, n = 10 neurons/4 mice; PPDA: 7.0 ± 0.4 pA vs. 6.5 ± 0.4 pA; *p* = 0.45, n = 5 neurons/3 mice Fig. 7c,d). However, administration of the antagonists of GluN2A (PEAQX) or GluN2B (Ro 25-6981) had no significant effect on either the frequency (PEAQX: 2.4 ± 0.5 Hz vs. 2.5 ± 0.5 Hz; *p* = 0.77, n = 5 neurons/3 mice; Ro 25-6981: 2.7 ± 0.3 Hz vs. 2.8 ± 0.4 Hz; *p* = 0.69, n = 7 neurons/4 mice) or the amplitude (PEAQX: 5.9 ± 0.5 Hz vs. 5.9 ± 0.6 pA; *p* = 0.65, n = 5 neurons/1 mice; Ro 25-6981: 7.7 ± 0.9 Hz vs. 7.6 ± 0.8 pA; *p* = 0.81, n = 7 neurons/4 mice) of the mEPSCs (Fig. 7a,b). These results indicate that GluN2C/2D may contribute to the facilitation of spontaneous presynaptic glutamate release.

## Discussion

In this study, we first proved the existence of GluN2C/2D in the ACC and compared its expression level with the hippocampus of adult mice by using western blot. Whole-cell patch-clamp results showed that bath perfusing the selective antagonist of GluN2C/2D, UBP145, blocked around 40% of the NMDAR mediated current in the pyramidal neurons of ACC as well as the hippocampal CA1. However, UBP145 did not alter the basic evoked synaptic transmission. GluN2C/2D did not participate in the synaptic plasticity. However, GluN2C/2D were involved in the spontaneous presynaptic release in the ACC. This study emphasized the significance of GluN2C/2D in the adult ACC, but has not unveiled the role of GluN2C/2D in the ACC under pathological conditions.

### Possible roles of GluN2C/2D in the synaptic transmission and plasticity in the ACC

As a voltage-dependent ligand-gated ion channel, NMDAR plays essential roles in the excitatory synaptic transmission as well as synaptic plasticity. The identity of the GluN2 subunits is critical in determining many biophysical and pharmacological properties. In the ACC of adult mice, previous studies have confirmed the existence of GluN1, GluN2A and GluN2B by using western blot. The GluN2A and GluN2B component in the NMDAR-mediated EPSCs have also been identified [18, 22]. In our study, besides detecting the expression of GluN2A and GluN2B, we further identified the expression and functional component of GluN2C/2D in the ACC. A few neurodevelopmental studies reported that in the neonatal brains, GluN2B and GluN2D subunits are highly expressed, and over the course of development, they are substituted or replaced by GluN2A and GluN2C in olfactory bulbs and cerebella. The GluN2D subunit is found exclusively in the diencephalon and brainstem compared with GluN2B [3, 23]. In the deep agranular frontal cortex, locally applied PPDA suppressed both intracortical and callosal evoked NMDAR-mediated responses[12]. Our study demonstrated the unneglectable status of GluN2C/2D in the ACC of adult mice.

We found that selectively inhibiting GluN2C/2D by UBP145 also blocked around 40% evoked NMDAR-mediated responses in the ACC. Here UBP145 is used at a relatively low concentration—below the published Ki's for GluN2A/2B and above that for GluN2C/2D[24]. Since UBP145 is a derivative of PPDA and displays a seven- to ten-fold selectivity for GluN2D-containing receptors over GluN2B- or GluN2A-containing receptors, it may be relatively selective. To further examine the proportion of GluN2C/2D, we blocked GluN2A and 2B currents first, and then examined the effect of UBP145. We found that the proportion of GluN2C/2D determined by this approach was 24.0%, smaller than applied UBP145 alone (37.9%). This may due to the possible side-inhibition effect of the UBP145 to the GluN2A/2B. Consistent with previous reports of the kinetic properties of GluN2C/2D[15], we found that the decay constant decreased when the GluN2C/2D antagonists were applied.

Our study proved that GluN2C/2D in the ACC did not participate in the synaptic plasticity. The application of UBP145 is not sufficient to block all the GluN2A/2B, which play important roles in NMDAR-dependent LTP/LTD. However, in other brain areas, GluN2C/2D was also reported to be involved in synaptic plasticity. In the hippocampus, PPDA at a higher dose was sufficient to block the induction of LTP[25]. It was reported that extrasynaptic GluN2D-containing NMDARs are recruited to the synapse during LTP of NMDAR-mediated EPSCs[14]. A positive allosteric modulator of GluN2C/2D-containing NMDARs, CIQ, rescued LTP in the striatum of a mouse model of Parkinson’s disease (PD). This study proposed GluN2D as a potential candidate for therapeutic intervention in PD [26].

### Presynaptic NMDARs modulate the presynaptic release

In addition to being expressed at the postsynaptic site, NMDARs are found in the presynaptic compartment. We also discovered presynaptic NMDARs by using a high dose AP-5 which reduced the frequency instead of the amplitude of mEPSCs in the ACC. This result is similar with other reports regarding presynaptic NMDARs in the entorhinal cortex [27, 28]. The subunit composition of presynaptic NMDARs varies according to brain regions and developmental stages. In our study, we applied selective antagonists for GluN2A, GluN2B and GluN2C/2D individually in the ACC, only the antagonists for GluN2C/2D inhibited the number of spontaneous responses. However, the inhibition of GluN2C/2D didn’t alter the evoked paired-pulse ratio at the interval of 50 ms. It is possible that glutamatergic inputs of evoked and spontaneous EPSCs are different. Currently, we can not evoke the same group of input fibers as spontaneous EPSCs do. GluN2C/2D may act through other pathways other than layer V-layer II/III. Furthermore, it is also possible that the presynaptic glutamate releasing pools in evoked and spontaneous procedures are distinct [29]. GluN2C/2D may only be involved in the spontaneous mechanisms.

In other investigations of presynaptic NMDARs, presynaptic NMDARs are mostly composed of di-heteromeric GluN1/GluN2A receptors at cerebellar parallel fiber–Purkinje cell synapses [30]. At mature cortical and hippocampal synapses, presynaptic NMDARs contain mostly GluN1/GluN2B receptors [31]. This discrepancy may be due to the cortical area differences and the precise location and function of presynaptic NMDARs however this is still under debate. Nevertheless, NMDAR containing GluN2C/2D subunits has been proved to mediate an increase in glutamate release at hippocampal CA3-CA1 synapses. Both the evoked, and spontaneous release, decreased when the activity of NMDARs containing GluN2B and GluN2C/2D subunits, but not GluN2A, was impeded[32]. The increase in glutamate release mediated by these NMDARs requires protein kinase A (PKA) activation. Future studies will track down the molecular mechanisms of the presynaptic modulation of GluN2C/2D in the ACC synapses.

### Physiological implications of GluN2C/2D

NMDARs are required for the synaptic plasticity associated with the mechanisms of learning and memory. Previous studies have shown that NMDARs in the ACC play important roles in pain perception and emotion regulation. Our present results indicate that GluN2C/2D in the ACC may also contribute to physiological and pathological brain functions. In the amygdala, GluN2C is reported to be involved in the acquisition and extinction of learned fear [5]. In addition, a positive allosteric modulator of GluN2C/2D, CIQ, reversed the MK-801-induced working memory deficit in spontaneous alternation in a Y-maze. CIQ also partially attenuated MK-801- and methamphetamine-induced hyperlocomotion and stereotyped behaviors. These behaviors are all positive and cognitive symptoms in schizophrenia [33]. Additionally, physiological experiments in rodents show that NMDAR antagonists generate oscillations by their action on the GluN2C-containing NMDARs that are prevalent in the thalamus. Such oscillations could contribute to symptoms of schizophrenia[34]. Therefore, future studies are clearly needed to study the physiological and pathological roles of the GluN2C/2D in the ACC, especially in learning memory and chronic pain.

## Data Availability

The datasets used and analyzed during the current study are available from the corresponding author on reasonable request.

## Abbreviations

NMDA: N-methyl-d-aspartate
NMDARs: NMDA receptors
ACC: Anterior cingulate cortex
UBP145: (2R*3S*)-1-(9-bromophenanthrene-3-carbonyl) piperazine-2,3-dicarboxylic acid
ACSF: Artificial cerebrospinal fluid
LTP: Long-term potentiation
post-LTP: Postsynaptic LTP
pre-LTP: Presynaptic LTP
LTD: Long-term depression
